# Critique on “Evaluation of Sex‐Based Differences in the Prescription of the Combination of Evidence‐Based Medicine After the Occurrence of an Acute ST‐Elevation Myocardial Infarction”

**DOI:** 10.1002/clc.70239

**Published:** 2026-01-26

**Authors:** Maiza Naseer, Sameer Haider, Touqeer Rehman

**Affiliations:** ^1^ Cardiology Department Gazi University Ankara Turkey

To the Editor,

Evelo et al. published an article entitled “Evaluation of Sex‐Based Differences in the Prescription of the Combination of Evidence‐Based Medicine After the Occurrence of an Acute ST‐Elevation Myocardial Infarction” which we read with interest [1]. The authors examined an important clinical question: Are women less likely than men to be discharged with the full combination of evidence‐based medicine (cEBM) after STEMI? In a retrospective cohort of 1467 patients in a teaching hospital in the Netherlands, they demonstrated that women were significantly less likely to be prescribed cEBM, particularly ACEi/ARB and statins. However, sex was not an independent predictor following multivariable adjustment [1]. This work is particularly important, especially in the context of studying a sizable real‐world cohort and establishing prescribing patterns in contemporary STEMI care. Sensitivity analyses for left ventricular ejection fraction and subgroup comparisons add further robustness to the work. More importantly, their study illustrates that even with guideline‐directed therapy, women continue to experience elevated rates of stroke and mortality when compared to men, reinforcing existing sex differences in cardiovascular care. There are some other limitations, not explicitly recognized by the authors, that should be considered when applying their findings in practice.

First, the single‐center design limits the generalizability of the findings. All patients were treated at a Dutch Teaching Hospital that offers a range of cardiology services, where the guideline treatment is often well followed. Prescribing patterns and patient characteristics will likely vary considerably in a community or rural hospital setting. As a result, the findings may not be generalizable to larger or more diverse populations [2]. Multicenter registries could help clarify if the finding that sex was not an independent predictor holds true across health care systems.

Second, medication prescriptions were measured only at the time of discharge, and there was no record of dose, intensity, or longitudinal adjustments. In the study, cEBM was defined operationally as a “recommendation” for prescription of all five drug classes on the day after the index discharge [1]. This binary definition totally ignores underdosing (e.g., low‐intensity statins, subtarget ACEi/ARB), titration, or discontinuation at a later date. Previous studies have demonstrated that underdosing and discontinuation, which are more frequently observed among women, are related to worse outcomes [3, 4]. By ignoring these variables, there is a risk that sex differences in secondary prevention will be underestimated.

Third, this study did not assess physician and system factors. The analysis was limited to patient‐level factors. However, cardiovascular care disparities are often a reflection of provider practice patterns, implicit bias, and institutional factors, such as discharge planning and outpatient follow‐up [5, 6]. Without accounting for these factors, it is difficult to know whether we are witnessing treatment gaps due to comorbidity rates between patients or structural inequities in care delivery.

Finally, adverse outcomes were described unadjusted for confounding factors. In this observational study, women experienced increased rates of 6‐month mortality and stroke, but those comparisons were not adjusted for age, baseline comorbidities, or treatment intensity. As evidenced in previous analyses, after full adjustments for factors, sex‐related mortality gaps tend to shrink [7]. Therefore, reporting unadjusted adverse outcomes may overstate the effects of sex itself, when risk factors and variables on care processes may be more impactful.

In moving this important research agenda forward, the following suggestions are warranted: (a) expand to multicenter cohorts to facilitate generalizability, (b) account for dose and longitudinal adherence, (c) explore provider and systems characteristics of prescribing, and (d) acknowledge confounders of adverse outcomes. These designs would better address whether observed disparities are biologically driven, patient‐related, or structural mediators.

In summary, Evelo et al. have provided evidence that women were less often prescribed cEBM after STEMI, while age, comorbidity, and anticoagulant use accounted for most differences. Given the heightened risk related to adverse events, a need arises prospectively, in multicenter settings, and with systems awareness, to start identifying systems to address inequities in post‐STEMI secondary prevention.

## Funding

The authors received no specific funding for this work.

## Ethics Statement

The authors have nothing to report.

## Consent

The authors have nothing to report.

## Conflicts of Interest

The authors declare no conflicts of interest.

## Data Availability

The authors have nothing to report.
